# Evidence of neuronal DNA damage in the brains of patients with Rett syndrome

**DOI:** 10.1242/dmm.052358

**Published:** 2025-09-01

**Authors:** Abril Morales, Elena Korsakova, Niloufar Mansooralavi, Peter Soliman, Sarvin Jahanbani, Michelle L. Olsen, Aparna Badhuri, William E. Lowry

**Affiliations:** ^1^ Molecular Biology Institute, University of California, Los Angeles (UCLA), Los Angeles, CA 90095, USA; ^2^ Department of Molecular Cell and Developmental Biology, UCLA, Los Angeles, CA 90095, USA; ^3^ Division of Dermatology, DGSOM, UCLA, Los Angeles, CA 90095, USA; ^4^ Broad Center for Regenerative Medicine, UCLA, Los Angeles, CA 90095, USA; ^5^ Jonsson Comprehensive Cancer Center, UCLA, Los Angeles, CA 90095, USA; ^6^ School of Neuroscience, Virginia Tech University, Blacksburg, VA 24061, USA

**Keywords:** Rett syndrome, DNA damage, MECP2, Single-nucleus RNA sequencing

## Abstract

Rett syndrome is characterized by the postnatal loss of neurophysiological function and regression of childhood development. Because the syndrome is X linked, and males with *MECP2* mutations generally do not survive birth, the study of this syndrome has been complicated by the fact that, in the female brain, a portion of neurons express wild-type *MECP2*, and another portion of neurons express a non-functional allele of *MECP2*. Here, we present an approach that enables transcriptional profiling of individual neurons and direct comparison of neurons that express functional *MECP2* with those that have diminished *MECP2* function. With this novel profiling approach, we found that mutant neurons from the brains of patients with Rett syndrome show patterns of defects in expression of synaptic and metabolic genes. A similar analysis of rat brain lacking MECP2 expression yielded similar patterns, suggesting that rat is a suitable *in vivo* model of Rett syndrome. These analyses also identified DNA damage and senescence transcriptional signatures specifically in MECP2-null neurons, suggesting a possible trigger of dysfunction in Rett syndrome. Together, these data highlight potentially defective molecular, physiological and metabolic pathways in brain neurons of patients with Rett syndrome.

## Teaser

**Summary:** Neurons lacking MECP2 show signs of elevated DNA damage and senescence transcriptional signatures, which could contribute to phenotypes in patients with Rett syndrome.

## INTRODUCTION

The disruption of the methyl-CpG-binding protein 2 (*MECP2*) gene, encoded on the X chromosome, is known to cause a severe neurodevelopmental disease called Rett syndrome (RTT) (Amir et al., 1999). It is an X-linked dominant disorder observed mostly in female heterozygotes, as males with this mutation on their only X chromosome typically fail to survive birth (Smrt et al., 2011; Cheung et al., 2012; Gogliotti et al., 2018). Female patients with RTT present with short stature overall, but have a relatively more profound microcephaly phenotype, suggesting a prominent role for *MECP2* in the brain (Xi et al., 2007; Pintaudi et al., 2010). Accordingly, although MECP2 protein is present in all tissues, expression of MECP2 is particularly high in all types of mature neurons of the central nervous system (Akbarian et al., 2001). Mice with brain-specific deletion of *Mecp2* phenocopy MECP2-null animals (Fyffe et al., 2008; Giacometti et al., 2007; Luikenhuis et al., 2004; Chen et al., 2001), and subsequent studies showed that neuronal-specific deletion of *Mecp2* recapitulated the phenotypes of MECP2-null animals (Fyffe et al., 2008; Samaco et al., 2009; Ito-Ishida et al., 2015; Ure et al., 2016).

MECP2 has been described as both a transcriptional stimulator and inhibitor, a regulator of RNA splicing, and a regulator of DNA methylation or reader of methylation (Tate et al., 1996; Nan et al., 1997, 1998a,b; Tudor et al., 2002; Young et al., 2005; Mellén et al., 2012). Moreover, loss of MECP2 has been proposed to lead to a variety of cell physiological defects, such as mitochondrial permeability, diminished dendritic branching, altered electrophysiological activity, and changes in nuclear and nucleolar size (Zhou et al., 2006; Farra et al., 2012). However, it is still not clear which of these observations are relevant for patients with RTT, and which should serve as proxies for experimentally targeting and developing therapeutic strategies. These observations have been made *in vitro* with cell-based models of the disease, in murine models of the syndrome lacking MECP2, in murine models with a gene replacement approach (Alvarez-Saavedra et al., 2007; Tao et al., 2009; Patterson et al., 2016; Goffin et al., 2011, 2014; Lamonica et al., 2017; Johnson et al., 2017), and in murine models that allowed for a cell type-specific analysis (Johnson et al., 2017). The identification of the phenotypes and gene expression changes that occur in human brain is critical to understand the etiology of the disease.

We previously created an isogenic *in vitro* system to model how the loss of MECP2 impacts development of human neural cell types by exploiting reprogramming of patient fibroblasts to a pluripotent state to create human induced pluripotent stem cells (hiPSCs) and differentiation towards particular neural lineages (neural progenitor cells and interneurons) (Ohashi et al., 2018). Our previously published work with this *in vitro* model of RTT indicated that specifically postmitotic neurons lacking MECP2 show defects in dendritic branching coincident with induction of P53 (also known as TP53), phosphorylated H2AX (pH2AX; a marker of DNA damage) and cellular senescence (Ohashi et al., 2018). Importantly, blocking senescence with P53 inhibition restored dendritic branching in neurons derived from patients with RTT (Ohashi et al., 2018). These results were consistent with those of the Galderisi group, who also showed senescence phenotypes in various models of RTT (Squillaro et al., 2008, 2010), opening up the possibility that defects in RTT are due to excessive P53 activity and/or senescence.

The study of brains of patients with RTT until very recently was limited to pathological examination of tissue or bulk molecular methods to understand the course of the disease. For example, Gogliotti et al. (2018) performed bulk RNA-sequencing analysis of a number of brain samples from patients with RTT versus those from unaffected controls and found hundreds of differentially expressed genes (DEGs). A re-analysis suggested upregulation of *P53* activity (Ohashi et al., 2018). However, the methods employed precluded identification of cell type-specific changes and was complicated by the fact that the brains of female patients with RTT are chimeric for both wild-type (WT) and mutant MECP2-expressing cells based on random X inactivation during development. Renthal et al. (2018) took advantage of differential single-nucleotide polymorphisms (SNPs) between maternal and paternal alleles coupled with single-cell RNA sequencing (sc-RNA-seq) to define gene expression changes in specific cell types. Although their study did identify DEGs in MECP2^−^ cells, the analysis did not implicate particular dysfunctional pathways to shed light on the effect of loss of MECP2 in neurons.

Here, we employed sc-RNA-seq for analysis of nuclei from brains of patients with RTT and unaffected controls to understand the effect of loss of MECP2 on neurons, compared to MECP2-expressing neurons in isogenic RTT brain as well as to WT brain, to determine which changes are caused directly by the loss of MECP2. In addition, we re-analyzed data from previous studies on RTT, as well as various *in vitro* models, to corroborate our findings and demonstrate similarities between human and rat models of RTT.

## RESULTS

To determine whether our previous results implicating DNA damage and senescence as an RTT phenotype is also found *in vivo*, we profiled neurons from the brains of patients with RTT. *MECP2* is an X-linked gene; thus, half of *MECP2* alleles are turned off in female brain owing to random X-chromosome inactivation, resulting in approximately half of the expressed alleles being WT and the other half harboring the *MECP2* mutation. By isolating cells from individuals, one can directly compare cells expressing the WT allele of *MECP2* to those expressing a mutant version. We hypothesized that any differences found between the two could be attributed solely to the mutation, because all cells come from the same genetic background and environment and because MECP2 is expressed constitutively across all neuronal cell types. Thus, age, lifestyle and other factors that typically contribute to molecular noise will be irrelevant in such comparisons because all the neurons studied will have experienced the same brain environment and only differ by expression of functional MECP2.

**Fig. 1.**
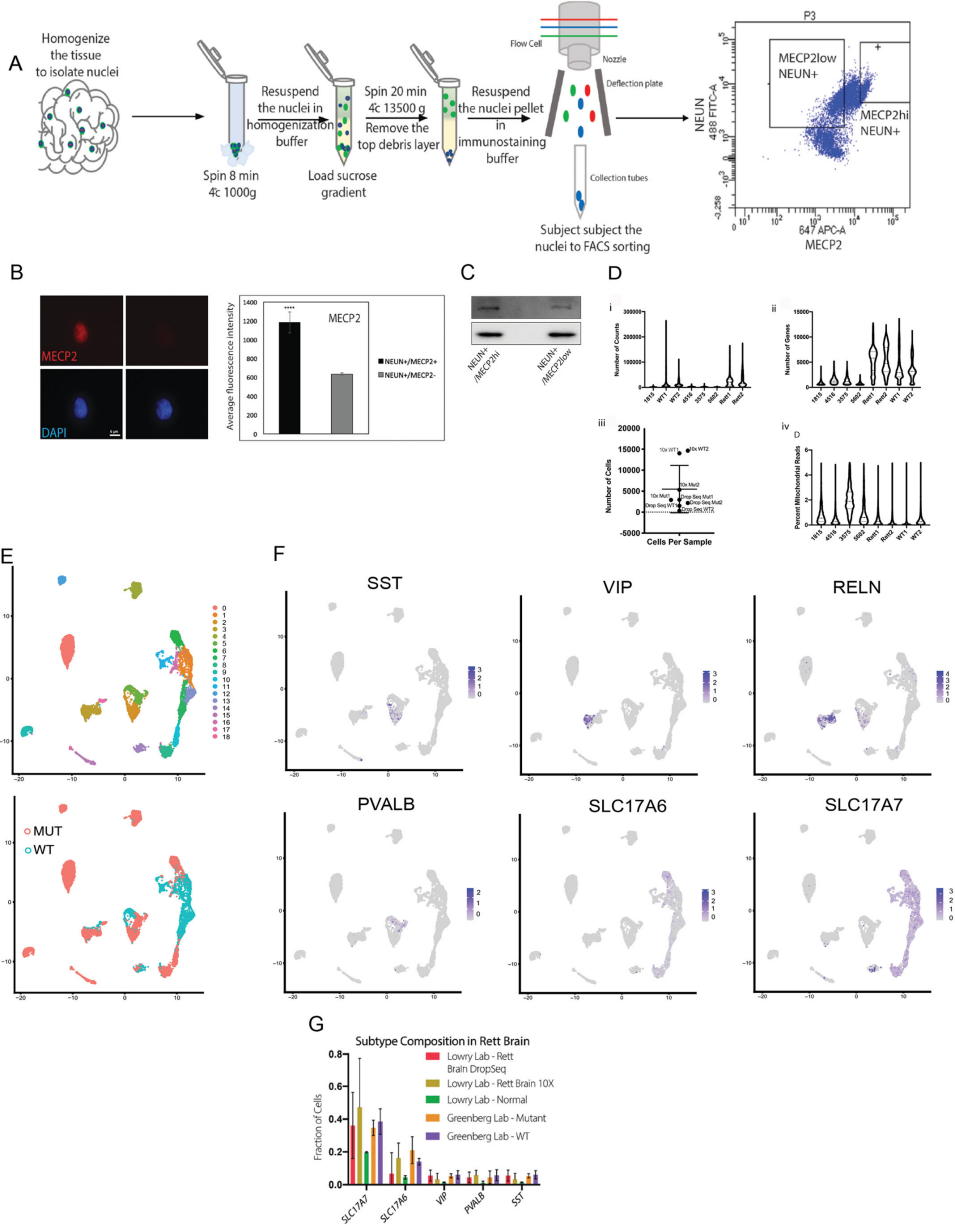
**A strategy to isolate wild-type and mutant MECP2-expressing neurons from human brain.** (A) The diagram depicts the process of nuclei isolation that employs sucrose gradient and several washes and centrifugations followed by immunostaining and fluorescence-activated cell sorting (FACS). (B) Left: immunostaining of the sorted MECP2-high and MECP2-low neuronal nuclei after cytospin of samples from brains of patients with Rett syndrome (RTT) shows a decrease in MECP2 expression in the MECP2-low fraction; images taken at 63×. Scale bar: 5 μm. We previously showed that the MECP2-high and -low populations represent wild-type MECP2-expressing versus mutant MECP2-expressing neurons (Ohashi et al., 2018). Right: quantification of immunofluorescence signal (*n*=100 per condition). Statistical analysis was performed using a paired two-tailed *t*-test; ****P<0.0001. (C) Western blot analysis verifying a decrease in MECP2 expression in the MECP2-low fraction isolated by FACS with an antibody against MECP2. (D) Quality control analysis of the single-nucleus RNA-sequencing (sn-RNA-seq) data: violin plots showing the number of counts per cell in the Drop-Seq and 10× RNA-sequencing data (i), number of genes per cell in each sample (ii), number of cells detected in each sample (iii) and percentage mitochondrial reads per cell in each sample (iv). (E) Uniform manifold approximation and projection (UMAP) plots of abundant neuronal subtypes as defined by the indicated markers, and UMAP showing 19 clusters identified during the RNA-sequencing analysis. (F) MECP2-low (MUT) and MECP2-high (WT) nuclei contribute to multiple identified clusters, expressing excitatory and inhibitory neuronal subtypes. (G) Proportions of neuronal subtypes in MECP2-high, MECP2-low and WT nuclei from three independent experiments (‘Lowry Lab’) and the data from Renthal et al. (2018) (‘Greenberg Lab’). Mutant, mutant MECP2-expressing nuclei from brains of patients with RTT; WT, wild-type MECP2-expressing nuclei from brains of patients with RTT; normal, wild-type nuclei from unaffected brains.

We took advantage of frozen postmortem brain samples from female patients with RTT to investigate differences in gene expression between MECP2^+^ and MECP2^−^ cells. We chose to isolate the nuclei from these sections because they tend to stay intact and preserve genetic material following brain tissue thawing (Fig. 1A). Although some *MECP2* mutations are known to lead to production of non-functional MECP2, we previously showed that the expression of *MECP2* in these particular samples was mosaic, suggesting that, for the patients samples used here, no functional protein was produced (Ohashi et al., 2018). Nuclei isolation was performed utilizing a sucrose gradient accompanied by a series of washes and centrifugations. The quality of nuclei isolation was assessed visually and quantitatively using a hemocytometer. We were particularly interested in the role of MECP2 in neurons, in which it is thought to be expressed at the highest level in the brain. Thus, we chose NeuN (also known as RBFOX3), a marker of mature neurons with nuclear localization, to isolate neuronal populations from MECP2^+^ nuclei. We stained the nuclei with MECP2, NeuN and 4′,6-diamidino-2-phenylindole (DAPI) and subjected them to fluorescence-activated cell sorting (FACS). We collected DAPI^+^/NeuN^+^ intact neuronal nuclei, which were either MECP2^+^ or MECP2^−^. It is important to note that, for all the samples used in this study, an MECP2-high and -low population was discernible in the NeuN^+^ pool of nuclei, confirming that MECP2 production was severely abrogated in a portion of the cells in each RTT brain analyzed here. To confirm that the nuclei sorting strategy was successful, we used cytospin, immunostaining and western blotting, which all confirmed that the expression level of MECP2 was highly diminished in the MECP2-low population of neurons (Fig. 1B,C).

WT and mutant neuronal nuclei were separately analyzed by both Drop-Seq (three individual samples from independent patients with RTT) and 10× RNA sequencing (two individual samples from independent patients with RTT) (quality control is described in Fig. 1D). After merging the datasets and controlling for batch effect, uniform manifold approximation and projection (UMAP) analysis generated 19 transcriptionally unique clusters in both the WT and mutant populations (Fig. 1E). Further analysis of the gene expression revealed several types of inhibitory and excitatory neuronal populations, consistent with expectations of a FACS isolation driven by NeuN expression.

This sorting and transcriptomic approach allowed for determination of whether the proportion of cell types was affected by MECP2 expression. Because NeuN expression tracked nearly identically with MECP2 expression – all 19 subtypes of neurons expressed NeuN and MECP2 to a similar degree – we were able to construct a proportional analysis of cell types between MECP2^+^ and MECP2^−^ neurons (Fig. 1F,G).

**Fig. 2.**
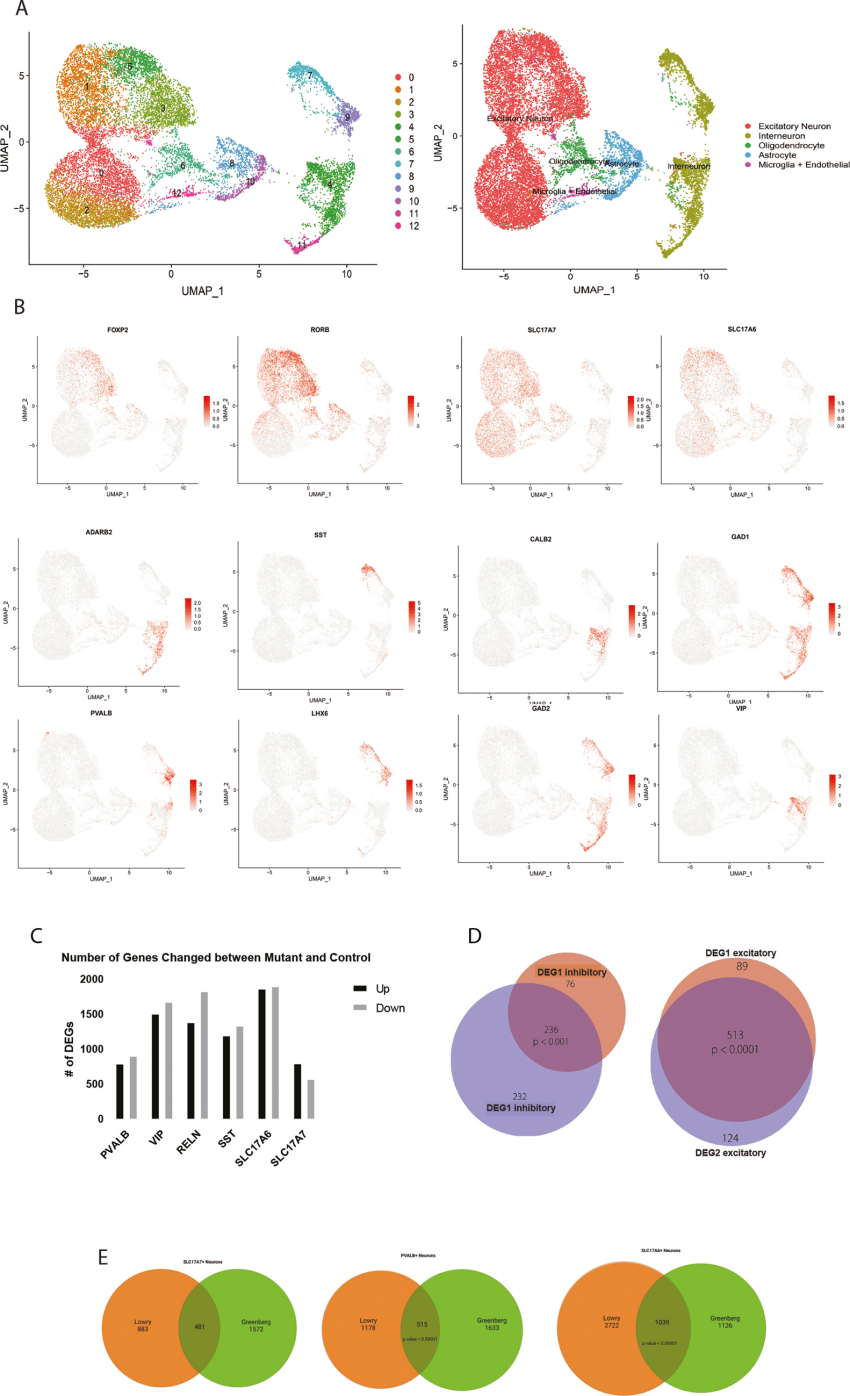
**Re-analysis of published sc-RNA-seq data.** (A) Left: UMAP plot showing 13 unique clusters identified during the re-analysis of the data from Renthal et al. (2018). Right: UMAP plot overlaying broad cell type categories. (B) UMAP plot highlighting cell subtypes defined by the expression of specific markers. (C) Number of genes changed in representative cell clusters. DEG, differentially expressed gene. (D) DEGs from three neuronal subtypes and their overlap between current data and the Renthal et al. (2018) dataset. (E) DEGs from three neuronal subtypes in the current study (‘Lowry’) and in Renthal et al. (2018) (‘Greenberg’) overlap significantly despite the fact that the methods employed and the tissue samples were highly distinct.

To confirm our hypothesis that neurons expressing the mutant allele of *MECP2* have lower expression of MECP2 protein, we re-analyzed data from the study by Renthal et al. (2018) (Goffin et al., 2011), which used SNP analysis to define WT versus mutant neurons from the brains of patients with RTT (Fig. 2). We then looked at DEGs in the two comparisons, hypothesizing that if the DEGs overlap significantly across the two approaches, the most likely explanation would be confirmation that reduced MECP2 levels correlated with mutant allele expression. We applied a *P*-value cut-off of <0.01 to the dataset to identify DEGs between MECP2^+^ and MECP2^−^ neurons from each brain (Fig. 2). In addition, the pattern of DEGs was conserved in both excitatory and inhibitory neurons in RTT brain (Fig. 2). We then compared DEGs identified in our own data versus those uncovered from the Renthal et al. (2018) data to identify overlap between the two methods (Fig. 2). In fact, DEGs from our 10× RNA-sequencing analysis had a robust, and statistically significant, overlap of >400 genes with DEGs from the SNP approach with independent brain samples despite the differences in methods (identification of WT versus mutant and sc-RNA-seq approaches) (Fig. 2) (Tao et al., 2009), validating our approach to isolate MECP2-high vs -low neurons from RTT brain. In addition, this analysis from eight RTT samples did not reveal a significant difference in the proportions of any neuronal subtypes among these brains from RTT patients (Fig. 1G). These results did not appear to be driven by batch effects or differential coverage in the sequencing depth (Fig. 1D).

**Fig. 3.**
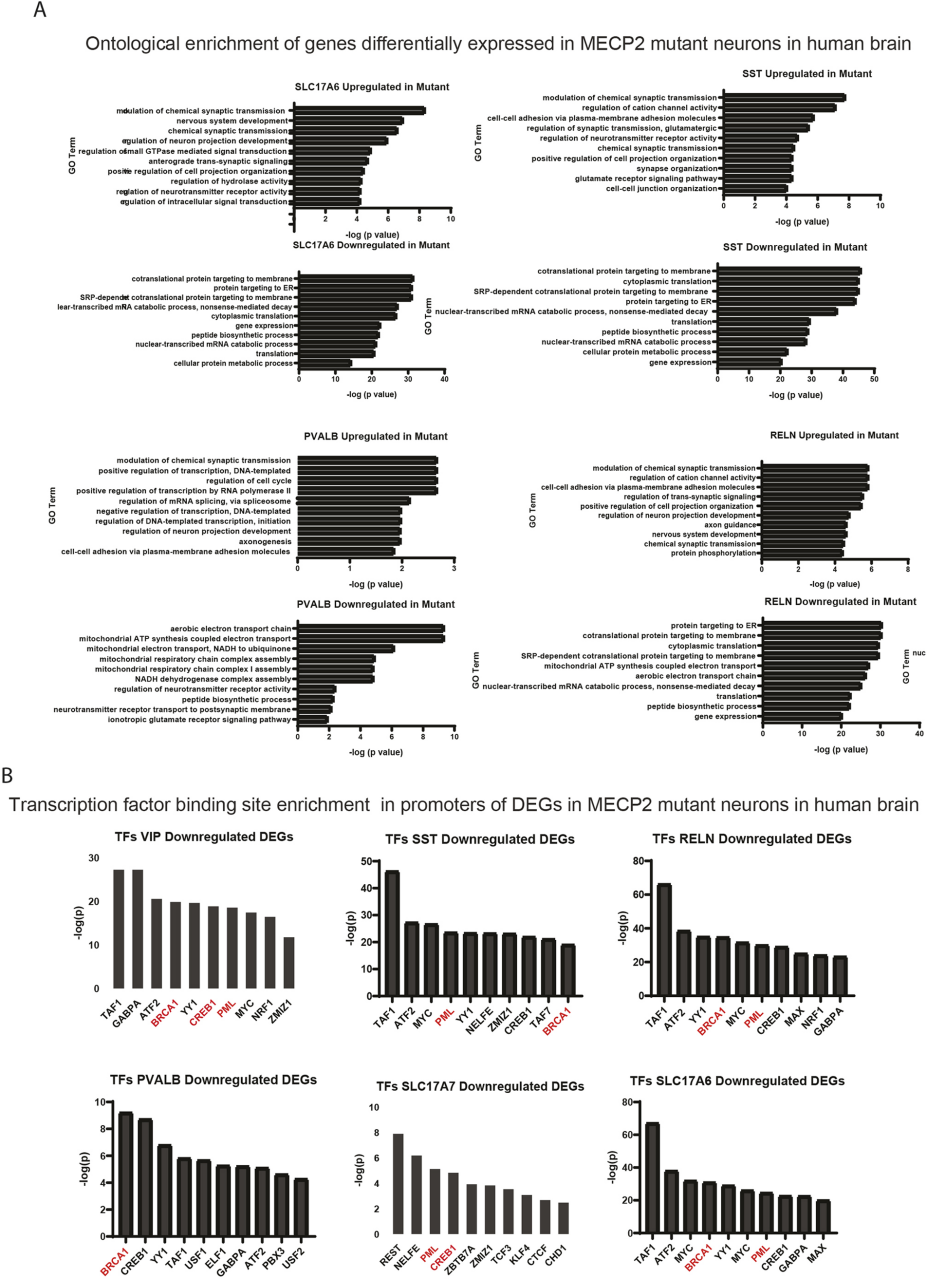
**Transcriptome analysis reveals alteration in synaptic and metabolic gene expression due to lack of MECP2 in human.** (A) Gene Ontology (GO) analysis of misregulated pathways indicates significant alterations in various pathways in inhibitory neurons and excitatory neurons in human brain. (B) Analysis of the transcription factor (TF) binding sites within the genes significantly downregulated owing to lack of MECP2 in excitatory and inhibitory neurons in human brain. Note that TFs related to neuronal stress and DNA repair are highlighted in red.

We then used Gene Ontology (GO) analysis to determine which biological or functional pathways are differentially regulated in neurons in the absence of MECP2 (Fig. 3). Remarkably, in the two major classes of excitatory neurons (SLC17A6^+^ and SCL17A7^+^), nearly all the gene expression changes induced in MECP2-low neurons appeared to be related specifically to neuronal physiology, such as ‘Regulation of Neuron Projection Development’, ‘Axonogenesis’, ‘Synapse Organization’, ‘Nervous System Development’, etc. (Fig. 3). This is an important observation considering recent data showing that neural organoids derived from patients with RTT also showed elevated levels of synapses and synaptic genes (Patterson et al., 2014). In the brains of patients with RTT, we also observed a large number of genes related to ribosomal function downregulated in at least one neuronal subtype, consistent with ribosomal dysfunction (Fig. 3A). Many of the downregulated gene expression changes appeared to be related to cellular metabolism, such as ‘Mitochondrial ATP Synthesis’, ‘Aerobic Electron Transport Chain’, etc. Considering the overlapping pattern of DEGs shown in Fig. 2, it is not surprising that the same GO analysis on inhibitory neuron subtypes (RELN^+^, SST^+^, VIP^+^) yielded similar results, in which the same categories of DEGs were found in both the upregulated and downregulated expression patterns (Fig. 3A). An analysis of enrichment of transcription factor (TF) binding sites in DEGs in various neuronal subtypes highlighted a conserved pattern of TFs across neurons, further suggesting that loss of MECP2 exerts a similar response in various types of neurons (Fig. 3B). In addition, enrichment for TFs such as BRCA1 is further evidence of DNA damage or DNA repair pathways acting in neurons lacking MECP2.

**Fig. 4.**
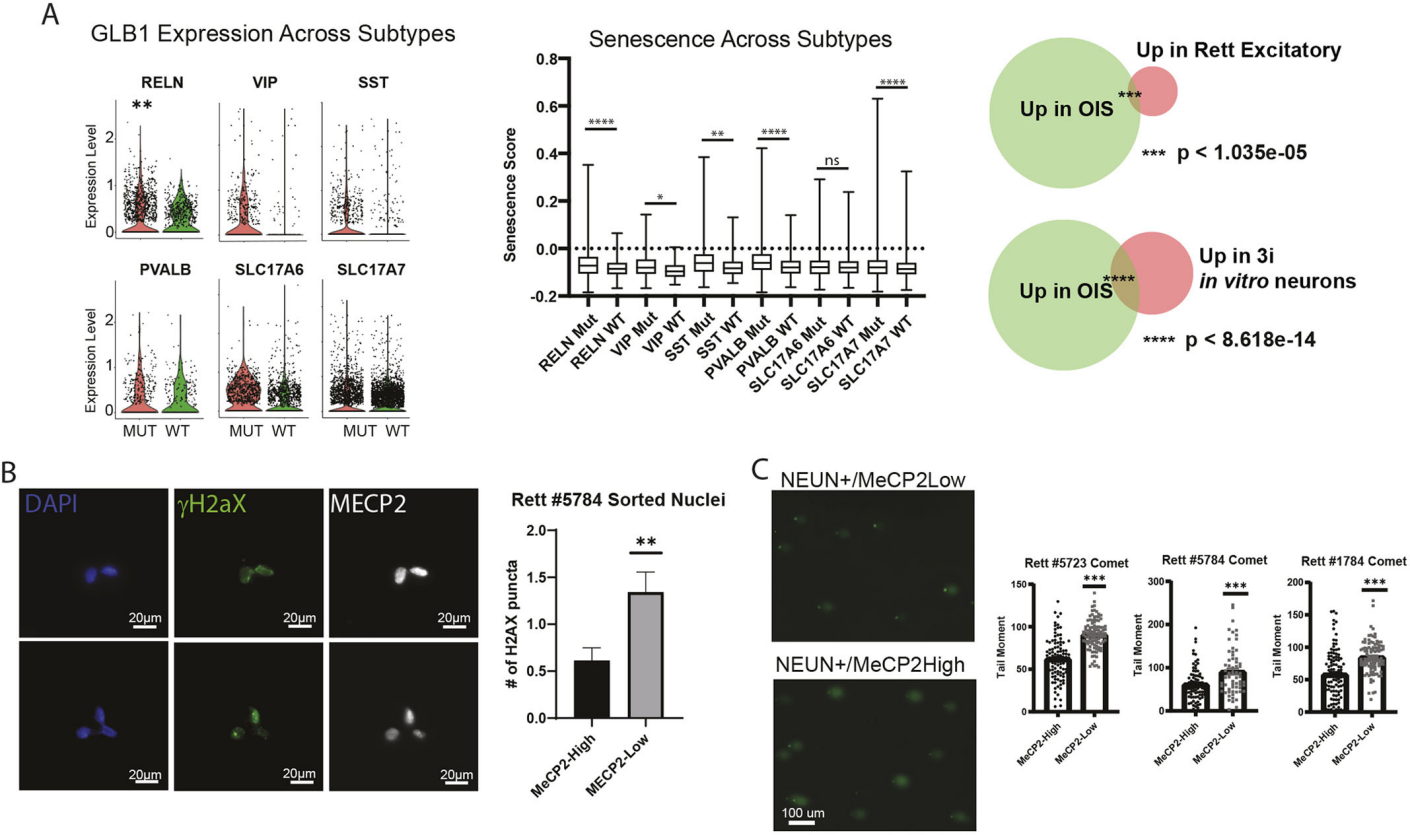
**Evidence of increased incidence of DNA damage in human brain neurons with MECP2 mutations.** (A) Left: measurement of *GLB1* gene expression as an indicator of senescent cells in mutant and wild-type MECP2-expressing neurons from brains of patients with RTT. Middle: box and whisker plot showing a senescence signature in wild-type and mutant MECP2-expressing neurons in six clusters by comparison of DEGs and established senescent transcriptomic signatures. Boxes represent the 25-75th percentiles, and the median is indicated; whiskers show range of expression of senescence signature genes. Right: Venn diagram showing the number of genes that overlap between excitatory mutant neurons from rat sn-RNA-seq experiments compared to oncogene-induced senescence (OIS) DEGs, as described previously (Morales et al., in press). 3i, three-inhibitor cocktail, as described in Chambers et al. (2009). Statistical analysis was performed using a hypergeometric distribution test (*n*=5 rat brain samples analyzed). (B) Left: immunostaining for phosphorylated (p)H2AX and MECP2 in human brain. Scale bars: 100 μm. Right: quantification of mean intensity of the immunostaining (*n*=100 cells per condition). (C) Left: comet assay on nuclei sorted from postmortem brains of patients with RTT. Three patient brain samples were used to fractionate nuclei based on NeuN and MECP2 expression and run the comet assay. In each case, the MECP2-low neurons showed longer comet tails, indicating increased DNA damage. Scale bars: 100 μm. Right: quantifications of results from similar experiments performed on brain samples from three patients with RTT. Statistical analysis was performed using paired two-tailed *t*-test; ns, not significant; *P<0.05, **P<0.01, ***P<0.001, ****P<0.0001.

Based on previous data showing elevated senescence in MECP2-null neurons *in vitro*, we probed more specifically for signatures of senescence in the RTT brain neurons lacking MECP2. First, we looked at RNA expression of *GLB1*, the gene that encodes β-galactosidase, the expression of which is a hallmark of senescence. In most subtypes, we indeed discovered that *GLB1* was expressed at a higher level in neurons lacking MECP2 (Fig. 4A, left). We next looked at a previously established senescence signature and found that most neuronal subtypes in the RTT brain showed elevated senescence in MECP2-low cells (Fig. 4A, middle). Another senescence signature also showed elevation in both *in vivo* and *in vitro* MECP2^+^ neurons, confirming these results (Fig. 4A, right).

To test more directly whether neurons in brains from patients with RTT have the same phenotype we observed *in vitro*, we first sorted nuclei using the same strategy as for RNA sequencing, and then stained for pH2AX, an established marker of DNA breaks, known to be elevated in MECP2-null neurons *in vitro* (Nan et al., 1998b; Young et al., 2005). With this approach, it was clear that MECP2-low neurons from brains of patients with RTT had increased numbers of pH2AX^+^ foci, consistent with increased DNA damage (Fig. 4B). As a more direct measure of DNA damage, we employed the comet assay, which measures the relative number of physical breaks in the genome through electrophoresis. Consistently, MECP2-low neurons from the brains of patients with RTT showed increased evidence of DNA breaks (Fig. 4C).

**Fig. 5.**
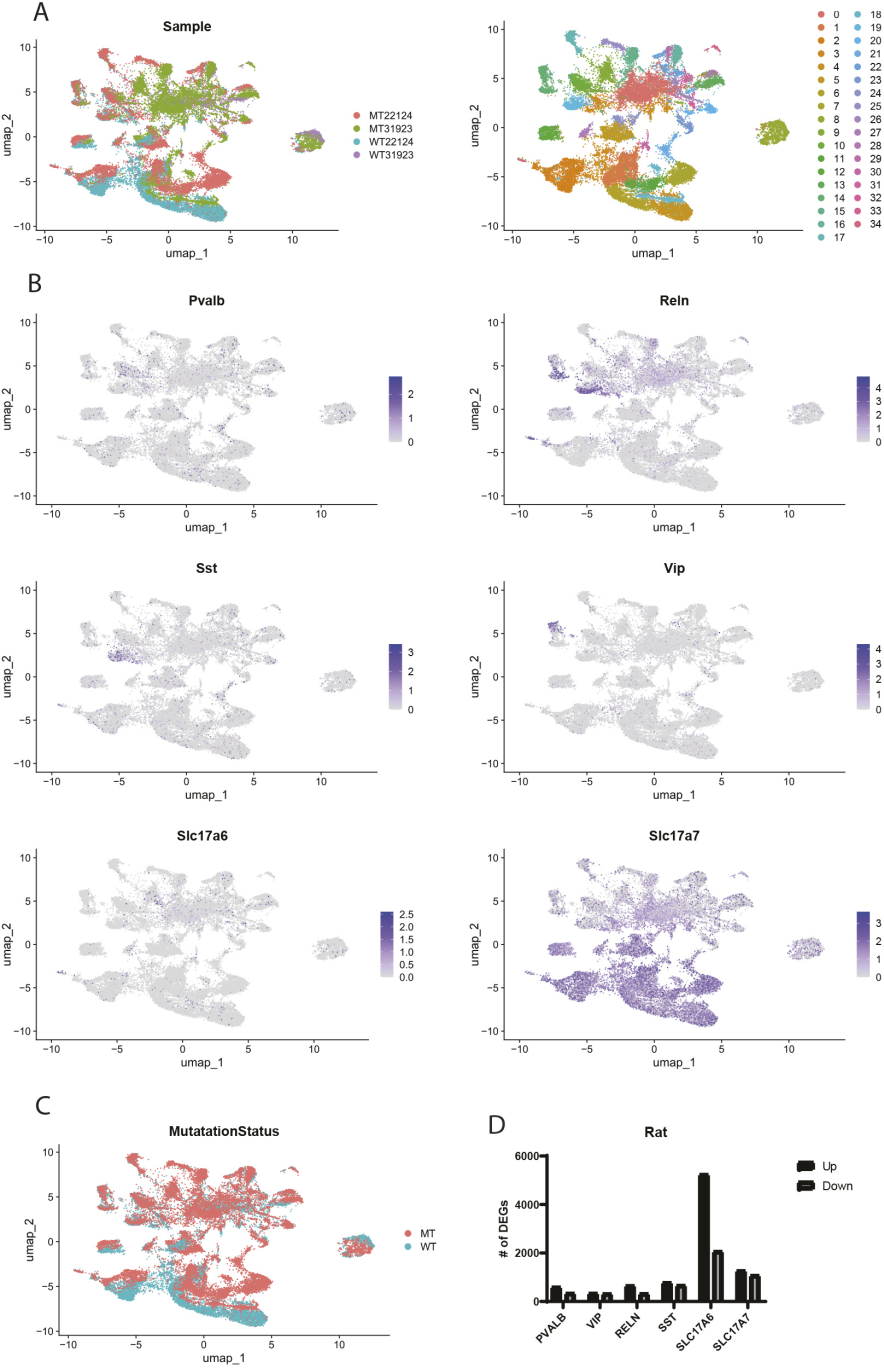
**sn-RNA-seq analysis of wild-type and RTT rat brain.** Nuclei were isolated from brains of wild-type rats and transgenic rats with loss of MECP2. (A) 10× single-cell RNA sequencing was performed, and single-cell profiles were obtained. (B) Gene expression profiles were plotted with UMAP, and several different neuronal subtypes were identified in various clusters as indicated. (C) A plot of DEGs between wild-type (WT) and RTT (MT) rat brain. (D) Plotted are the number of DEGs (upregulated and downregulated) in each neuronal subtype.

**Fig. 6.**
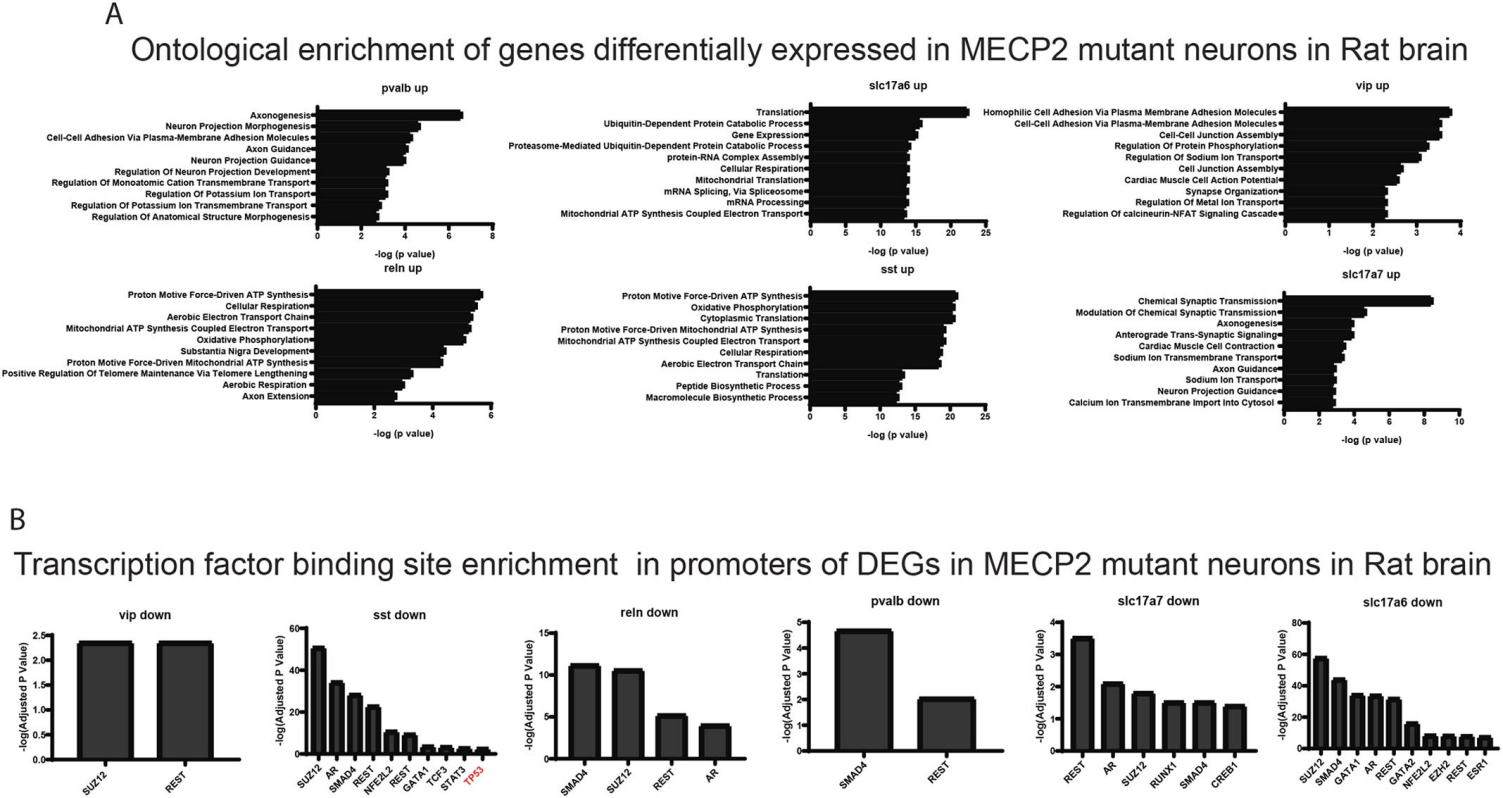
**Transcriptome analysis reveals alteration in synaptic and metabolic gene expression due to lack of MECP2 in rat.** (A) GO analysis of misregulated pathways indicate significant alterations in various pathways in inhibitory neurons and excitatory neurons in rat brain (bottom). (B) Analysis of the TF binding sites within the genes significantly downregulated due to lack of MECP2 in excitatory and inhibitory neurons in rat. Note that TFs related to neuronal stress and DNA repair are highlighted in red.

To further extend this paradigm to a model system amenable to experimental manipulation, we assessed whether MECP2-null neurons in the rat brain also have elevated senescence and/or DNA damage. We started by performing single-nucleus RNA sequencing (sn-RNA-seq) on brain tissue from a rat model of RTT. In this case, we analyzed all nuclei isolated and identified 35 individual cell types across WT and transgenic brains (Fig. 5A). We did observe a large number of gene expression changes in the MECP2-null brain across all cell types (Fig. 5B,C). There was a general imbalance towards upregulation of genes in the rat brain due to loss of MECP2, especially in one excitatory neuronal subtype, unlike in human brain, in which the DEGs were balanced in number across subtypes (Fig. 5D). Probing the nature of the DEGs, the majority of the ontological categories related to synapse function, mitochondrial activity and ribosomal dysfunction across all neuronal subtypes were detected (Fig. 6A). Again, TF binding site enrichment in DEGs of the RTT rat brain showed similar TFs across neuronal subtypes, which were also similar to those identified in DEGs from the brains of patients with RTT, such as SMAD4, SUZ12, REST, CREB1, etc. (Fig. 6B).

**Fig. 7.**
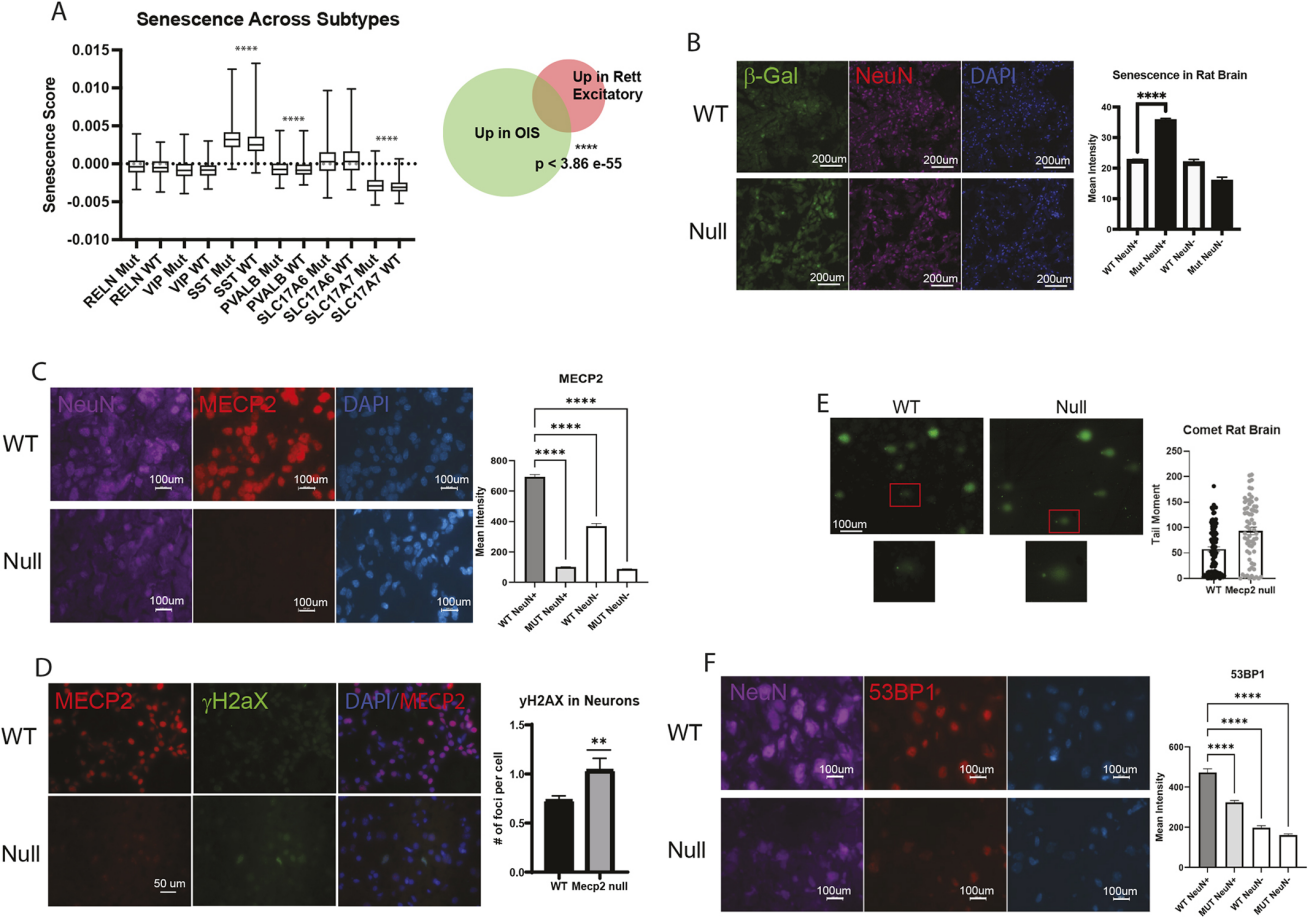
**Evidence of senescence and DNA damage in rat brain lacking MECP2.** (A) Left: probing single-cell gene expression data from rat brain for a signature of senescence highlights an increase in SST^+^, PVALB^+^ and SLC17A7^+^ neurons lacking MECP2. Boxes represent the 25-75th percentiles, and the median is indicated; whiskers show range of expression of senescence signature genes. Right: applying the same approach across all excitatory neurons with an OIS signature also showed a statistically significant effect. (B) *In situ* immunofluorescence analysis of β-galactosidase (β-Gal) showed an overlap between a neuronal marker (NeuN) and elevated senescence. Scale bars: 200 μm. (C) Immunostaining transgenic and wild-type rat brain for MECP2 and NeuN confirmed the deletion of MECP2 in the transgenic mice. Scale bars: 100 μm. (D) Co-immunostaining of MECP2 and pH2AX shows elevated pH2AX staining in MECP2-null neurons. Scale bar: 50 μm. (E) Comet assay on cells isolated from wild-type or MECP2-null rat brain shows increased tails in the absence of MECP2, indicating more DNA damage. Scale bar: 100 μm. (F) Co-immunostaining of MECP2 and P53BP1, another marker of DNA damage, shows that P53BP1 is diminished in neurons of MECP2-null brain. Scale bars: 100 μm. Quantifications of staining patterns were performed on multiple images (B-F, right). Statistical analysis was performed using two-way ANOVA; **P<0.01, ****P<0.0001.

To uncover potential neuronal senescence in RTT rat brain, we again probed for signatures of senescence in the sn-RNA-seq data and found that MECP2-null neurons showed elevated senescence in SST^+^, PVALB^+^ and SLC17A7^+^ neurons (Fig. 7A). Measuring more broadly across all excitatory neurons, this signature again proved to be elevated in particular neuronal subtypes as in human brain (Fig. 7A). As a more direct measure, we used β-galactosidase activity *in situ* analysis and found an elevated level of senescence in mutant neurons in the rat brains (Fig. 7B). Immunostaining for MECP2 and pH2AX provided compelling evidence of elevated DNA damage in the absence of MECP2 (Fig. 7C,D), as was shown in the brains of patients with RTT (Fig. 4). To confirm this observation, a comet assay on brain tissue from WT versus MECP2-null rat brain also showed elevated DNA damage in the absence of MECP2 (Fig. 7E). Finally, staining for P53BP1 (also known as TP53BP1), another protein known to localize to sites of DNA damage, showed diminished expression in neurons lacking MECP2 (Fig. 7F). Previous work has shown that the combination of elevated pH2AX and decreased P53BP1 indicates a cell state in which DNA repair is deficient and thus P53BP1 is not recruited to sites of DNA damage (Sabin et al., 2019).

## DISCUSSION

This study sheds new light on the etiology of RTT by molecular interrogation of individual neurons from brain tissue of patients with RTT. From these data, several important observations were made about selective susceptibility of individual subtypes of neurons, the proportions of neuronal subtypes, effects on expression of synaptic genes, and the nature of stress in neurons induced by loss of *MECP2.* Given their critical role in cognition, it is especially important that neurons are equipped to withstand various threats such as injury and disease. This is especially true for postmitotic cells, which bear special relevance to RTT because MECP2 is most highly expressed in these cells specifically (Dastidar et al., 2012). It is known that many forms of neuronal stress response lead ultimately to the activation of various TFs, which in turn act to determine cell fate. Some hypothesize that the resulting transcriptional changes are a cellular attempt to mitigate the initial stress or its downstream impacts, remove damaged neurons or alter brain activity to compensate for the stressor, or are even a direct pathological symptom (Farley and Watkins, 2018). Further, neuroinflammation, a known consequence of chronic stress (Solleiro-Villavicencio and Rivas-Arancibia, 2018), has been linked to MECP2 mutations as measured by observed dysregulation of several acute-phase response proteins in a mouse model of RTT (Cortelazzo et al., 2017). The current study attempted to define which types of neurons in the RTT brain are undergoing stress and the potential molecular or physiological triggers of that stress.

Studies from our group and others have suggested an important role of senescence in RTT. It has been argued that senescence may be a cellular adaptation designed to conserve energy required for division and differentiation so the cell can survive when facing stress (Ishikawa, 2006). The Galderisi group has published several studies demonstrating senescence in mesenchymal stem cells (MSCs) from patients with RTT, partially MECP2-silenced human MSCs, MECP2-silenced human neuroblastoma cells, and heterozygous MECP2 mutant mouse mesenchymal stromal cells and neural stem cells (Squillaro et al., 2010, 2008, 2019; Mucerino et al., 2017; Alessio et al., 2018). However, in our previous study on hiPSCs derived from patients with RTT, we found senescence associated secretory phenotype (SASP) induction in MECP2-null interneurons, consistent with these previous results (Ohashi et al., 2018).

Our data suggest that MECP2-null neurons could have dysfunctional mitochondrial metabolism, which is consistent with the results from previous studies (Alessio et al., 2018; Squillaro et al., 2019). A functional exploration of this observation can only really be carried out with an *in vitro* model of RTT, and this will be described elsewhere (Morales et al., in press). This is interesting in light of what is known about mitochondrial diseases caused by mutations in genes for mitochondrial proteins. These diseases share many of the characteristic clinical symptoms of RTT, including intellectual disability, motor problems and seizures (Chinnery and Turnbull, 2000; Chinnery et al., 2001). In addition, they are accompanied by evidence of oxidative damage and increased blood lactate and pyruvate content (Lee et al., 2014), which have also been identified as features of RTT (Haas et al., 1995).

There is also extensive evidence of the role of abnormal mitochondria in oxidative stress, a type of neuronal stress heavily implicated in many diseases including RTT, which leads to the aforementioned oxidative damage. Oxidative stress is a cellular condition caused by excessive reactive oxygen species (ROS). This therefore suggests a positive feedback mechanism in which mitochondrial defects (initially stemming from MECP2 loss) lead to overproduction of ROS, which in turn causes further mitochondrial defects due to oxidative stress. This cycle may explain the delayed onset of RTT as the initial mitochondrial deficiency caused by loss of MECP2 might not produce a large enough effect to lead to symptoms, but accumulation of mitochondrial defects over time can. However, this hypothesis conflicts with the fact that, unlike many other neurological diseases in which oxidative stress is implicated (such as Alzheimer's disease and Parkinson's disease) (Farley and Watkins, 2018), RTT is not degenerative. Additionally, reintroduction of MECP2 into MECP2-null and -deficient symptomatic mouse models rescued WT levels of several oxidative stress markers (Shulyakova et al., 2017). One possible explanation for this is that once the initial driver of mitochondrial dysfunction is ameliorated, the brain is able to correct for the accumulation of mitochondrial defects, likely through the major mitochondrial DNA repair mechanism, base excision repair (Massimino et al., 2005). However, further studies are required to determine whether this is true, and more generally how the dynamics of cellular stress and cellular stress response work together to produce RTT symptoms without typical neurodegeneration.

Another question not answered by the current study is exactly how loss of MECP2 function can lead to metabolic dysfunction. Although MECP2 is known to bind methylated DNA (Meehan et al., 1992), it is also now clear that MECP2 can regulate RNA as well (Young et al., 2005; Cross et al., 1994), and this protein has been implicated in a panoply of different functions. One could speculate that loss of function of an epigenetic regulator such as MECP2 could disrupt gene regulation of important metabolic proteins or perhaps induce a stress response that leads to P53 upregulation and subsequent diminishment of metabolic activity in an active effort to diminish ROS levels and allow repair. Or, many have argued that MECP2 can regulate gene expression as a DNA-binding protein or, more recently, through its ability to directly bind RNA (Squillaro et al., 2019; Shulyakova et al., 2017; Can et al., 2019; Enikanolaiye et al., 2020). Finally, it is tempting to speculate that because epigenetic regulation and metabolism share similar substrates and products, perhaps epigenetic regulation in the nucleus leads to an imbalance of substrates available for metabolic enzymes and therefore metabolic disruption. Future efforts will exploit *in vitro* models of RTT to test these hypotheses.

Finally, this study is the first to profile and compare human and rat models of RTT. The fact that both models showed elevated DNA damage and senescence validates previous *in vitro* studies, but more importantly points to an important molecular phenotype that could certainly be causative for human symptoms of RTT. Because of the availability of the rat model and its similarity to patients with RTT, it is important to highlight the utility of this model for both behavioral and molecular phenotyping.

## MATERIALS AND METHODS

### Human brain tissue from patients with RTT

Human tissue transcriptomic profiling was performed from five individual cortical tissue samples from patients with RTT. Individual tissue samples were collected from the cortical brain repository at the National Institutes of Health BioBrainBank.

### Animals

*Mecp2^tm1.1Bird^*/Y (*Mecp2*/Y, which are *Mecp2*-null) and age-matched WT (+/Y) rats were used in animal studies. Generation of this model is described in more detail in Enikanolaiye et al. (2020) and consisted of crosses of *Mecp2*^ZFN/+^ females (SD-Mecp2tm1sage) to WT S100b eGFP to produce *Mecp2*^ZFN/y^ and *Mecp2*^ZFN/+^ rats.

### Nuclei isolation from frozen brain samples

Nuclei were isolated as described previously (Allison et al., 2021). Briefly, brain tissue was cut on ice using a scalpel and homogenized in a glass dounce. The homogenate was then filtered through a 40-μm cell strainer and centrifuged at 1000 ***g*** for 8 min at 4°C. The pellet was resuspended in a homogenization buffer and mixed with equal amounts of iodixanol. This mixture was then gently placed in a new tube over 29% iodixanol. Nuclei were centrifuged at 13,500 ***g*** for 20 min at 4°C. The pellet was resuspended in the immunostaining buffer and incubated for 15 min at 4°C. Next, primary antibody was added to the nuclei pellet and incubated on a rotator at 4°C for 40 min. The following primary antibodies were used: rabbit anti-MECP2 (Diagenode, C15410052, 1:250) and chicken anti-NeuN (Millipore Sigma, MAB377, 1:250). Nuclei were centrifuged at 400 ***g*** for 5 min at 4°C and washed with PBS/bovine serum albumin twice. Secondary antibodies conjugated with Alexa Fluor 488 and 594 (Life Technologies, A-21202 and A-21203, respectively, 1:500) were added, accompanied by DAPI (Invitrogen, 1:500), and the nuclei were incubated on a rotator at 4°C for 30 min. Immunostained nuclei were subjected to FACS.

### Rat brain samples

RTT rat brains were provided by Dr Michelle Olsen, Virginia Tech University. Brains were embedded in OCT for sectioning and immunostaining and used to generate single-cell suspensions for comet assays. Frozen brain portions were used to create nuclear preparations followed by 10× Illumina sequencing as described above for human brain.

### Library preparation and sequencing

Sorted nuclei (sn-RNA seq) or isolated RNA (bulk RNA sequencing) were delivered to the UCLA Technology Center for Genomics and Bioinformatics, where libraries for RNA sequencing were prepared. The samples were sequenced using a NovaSeq 6000 S2 PE 2×50 with 50,000 reads per cell.

### sc-RNA-seq analysis

Fastq files were aligned using Cellranger version 8.0.0. Aligned counts were clustered using Seurat v3 and the standard clustering pipeline and parameters. No batch correction was performed. Quality control measures were used to remove any genes in fewer than three cells. No additional ambient RNA or doublet finder approach was used. Cells were removed for quality control if they contained fewer than 500 genes per cell or more than 5% of their gene counts associated with mitochondrial reads. No ceiling for the number of genes per cell was used for quality control. Normalization occurred using the NormalizeData() command in Seurat v3 with the default LogNormalization and a scale factor of 10,000 (all default). Scaling was performed using the ScaleData() command across all genes, but principal component analysis was performed with the RunPCA() command on the top 2000 variable genes identified with the FindVariableFeatures() command using the default vst method. The top ten principal components were used for clustering and UMAP calculation, with a resolution of 0.5 used to define clusters. In addition to using these clusters, cells were also classified by their marker identity of *SLC17A6*, *SLC17A7*, *RELN*, *PVALB*, *SST* and *VIP*. To do this, cells with more than one count of that gene marker were classified into groups for some of the downstream analyses. Thus, when cells, clusters or annotations are marked by one of these six marker gene subsets, it was calculated by putting cells into bins based upon a minimum of one count for the marker gene. In other cases, the clusters were used – the distinctions are annotated in the figure panels. In both cases, cluster markers were calculated using the Wilcoxon rank sum test to compare distributions across individual clusters and the rest of the population. The input file had the GO term, *P*-value and q-value downloaded from Enrichr.

### Senescence signatures

Senescence gene programs were obtained from published datasets (The TP53 Database). Gene programs were evaluated in each condition with a module eigengene calculation implemented from the WGCNA package. Significance was evaluated using two-way ANOVA. Violin plots for *GLB1* expression were generated based upon single-cell analysis, using Seurat v3.

### Confocal analysis

The cells were analyzed by confocal microscopy using an inverted confocal laser microscope, Zeiss LSM880 with Airyscan, at 100×.

### Western blotting

Cell lysate was prepared using RIPA buffer (Pierce) supplemented with Halt Protease Inhibitor Cocktail (Thermo Fisher Scientific) and Halt Phosphatase Inhibitor Cocktail (Thermo Fisher Scientific). Total protein concentration was determined using a BCA Protein Assay Kit (Thermo Fisher Scientific) following the manufacturer's protocol. Equal protein concentrations were loaded onto NuPAGE 4-12% Bis-Tris gel (Thermo Fisher Scientific) and run at 150 V for 90 min in running buffer, containing 25 ml of 20× NuPAGE MOPS SDS Running Buffer (Thermo Fisher Scientific) and 475 ml Milli-Q water. Next, protein was transferred onto the nitrocellulose membrane at 30 V for 60 min in transfer buffer, containing 25 ml of 20× NuPAGE Transfer Buffer (Thermo Fisher Scientific), 100 ml methanol (Thermo Fisher Scientific) and 375 ml Milli-Q water. The membrane was blocked overnight at 4°C in OneBlock Western-FL Blocking Buffer (Genesee Scientific), then incubated in the primary antibody at 4°C overnight. The following primary antibodies were used: rabbit anti-MECP2 (Diagenode, C15410052, 1:1000), rabbit anti-histone H3 (Abcam, ab1791, 1:1000) and mouse anti-β-actin (Santa Cruz Biotechnology, sc-47778, 1:500). The membrane was washed twice with 0.1% PBST and incubated in anti-rabbit or anti-mouse horseradish peroxidase-labeled secondary antibody (Thermo Fisher Scientific, 31460 and 31430, respectively, 1:100,000) for 1 h at room temperature. The membrane was washed twice with 0.1% PBST, and SuperSignal West Femto Maximum Sensitivity Substrate (Thermo Fisher Scientific) was added to the membrane before it was subjected to film exposure.

### Immunofluorescence and image quantification

Frozen OCT tissue sections were washed with PBS and fixed with 4% paraformaldehyde (Electron Microscopy Sciences) for 15 min at room temperature. Next, the cells were washed with 0.1% PBST three times and blocked in MAXblock Blocking Medium (Active Motif) for 1 h at room temperature, then incubated overnight at 4°C in the primary antibody. The following primary antibodies were used: rabbit anti-MECP2 (Diagenode, C15410052, 1:1000) and mouse anti-pH2AX (Millipore, 05-636, 1:2000). Next, the slides were washed three times with 0.1% PBST and secondary antibody conjugated to Alexa Fluor 488, 568, 594 or 647 (Life Technologies, A-21202, A-21207, A-21203 and A-31571, respectively, 1:500), was used, accompanied by DAPI (Invitrogen, 1:500). Slides were then washed three times with 0.1% PBST and mounted using Prolong Gold (Invitrogen). Mean fluorescence intensity and/or puncta number per cell were quantified using ImageJ.

### β-Galactosidase senescence assay

β-Galactosidase senescence assay was performed using either the Senescence β-Galactosidase Staining Kit from Cell Signaling Technology or CellEvent™ Senescence Green Detection Kit from Invitrogen following the respective manufacturer’s protocol. The number of blue/green cells and number of total cells were quantified using the Cell Counter plugin in ImageJ.

### Alkaline comet assay

The alkaline comet assay was performed as previously described (Maiuri et al., 2017), with some minor changes. From a 24-well tissue culture plates (Greiner), cells were harvested with TrypLE, spun down and resuspended in 0.5% low-melting-point agarose at 37°C in a 1:10 ratio. The cell suspension was spread onto agarose-coated slides and allowed to polymerize for 20 min in the dark. After agarose solidification, samples were incubated in lysis buffer (10 mM Tris-HCl, pH 10, 2.5 M NaCl, 0.1 M EDTA, 1% Triton X-100) for 2 h. Sorted nuclei from the brain were incubated in modified lysis buffer (1 mM Tris-HCl, pH 10, 2.5 M NaCl, 0.1 M EDTA, 1% Triton X-100) for 1 h. Following removal of lysis buffer, samples were incubated in alkaline running buffer (0.3 M NaOH, 1 mM EDTA) for 30 min and finally electrophoresed at 300 mA for 30 min at 4°C. Slides were washed three times with distilled water (dH_2_O) and fixed with cold 70% ethanol. Cells were then stained with Vista Green DNA staining solution (Abcam) for 15 min at room temperature, washed with dH_2_O and left overnight for agarose to dry. Images were acquired on a Zeiss Axio Imager A2 and analyzed using CometScore 2.0 software.
